# Prehabilitation for patients with colorectal cancer: a snapshot of current daily practice in Dutch hospitals

**DOI:** 10.1186/s13741-023-00299-y

**Published:** 2023-05-08

**Authors:** Charlotte J. L. Molenaar, Muriël Reudink, Charissa R. Sabajo, Loes Janssen, Rudi M. H. Roumen, Joost M. Klaase, Gerrit D. Slooter

**Affiliations:** 1 Department of Surgery, Máxima Medical Centre, Veldhoven, the Netherlands; 2 Department of Surgery, Maastricht University Medical Centre, Maastricht, the Netherlands; 3 Department of Surgery, University Medical Centre Groningen, Groningen, the Netherlands

**Keywords:** Multimodal prehabilitation, Preoperative screening, Surgery, Colorectal cancer

## Abstract

**Background:**

Multimodal prehabilitation programmes are increasingly being imbedded in colorectal cancer (CRC) pathways to enhance the patient’s recovery after surgery. However, there is no (inter)national consensus on the content or design of such a programme. This study aimed to evaluate the current practice and opinion regarding preoperative screening and prehabilitation for patients undergoing surgery for CRC throughout the Netherlands.

**Methods:**

All regular Dutch hospitals offering colorectal cancer surgery were included. An online survey was sent to one representative colorectal surgeon per hospital. Descriptive statistics were used for analyses.

**Results:**

Response rate was 100% (*n* = 69). Routine preoperative screening of patients with CRC for frailty, diminished nutritional status and anaemia was the standard of care in nearly all Dutch hospitals (97%, 93% and 94%, respectively). Some form of prehabilitation was provided in 46 hospitals (67%) of which more than 80% addressed nutritional status, frailty, physical status and anaemia. All but two of the remaining hospitals were willing to adopt prehabilitation. The majority of the hospitals offered prehabilitation to specific subgroups of patients with CRC, such as the elderly (41%), the frail (71%) or high-risk patients (57%). There was high variability in the setting, design and content of the prehabilitation programmes.

**Conclusions:**

Whereas preoperative screening is sufficiently incorporated in Dutch hospitals, standardised enhancement of the patient’s condition in the context of multimodal prehabilitation seems to be challenging. This study presents an overview of current clinical practice in the Netherlands. Uniform clinical prehabilitation guidelines are vital to diminish heterogeneity in programmes and to produce useful data to enable a nationwide implementation of an evidence-based prehabilitation programme.

**Supplementary Information:**

The online version contains supplementary material available at 10.1186/s13741-023-00299-y.

## Background

Preoperative risk screening prior to surgery is standard of care in the Netherlands. Once surgery is indicated, patients are referred to the preoperative screening outpatient clinic to be evaluated on general health under supervision of anaesthesiologists. Adequate screening is crucial to identify deficiencies. The interventions prescribed in this setting should be distinguished from the concept of prehabilitation. Prehabilitation not only addresses deficiencies but also refers to interventions in the preoperative period to improve overall functional capacity prior to surgery and consequently improve outcome and allow quicker recovery postoperatively (Minnella and Carli 2018). It has been shown to reduce length of hospital stay (Santa Mina et al. 2014) and complication rate (Barberan-Garcia et al. 2018; Minnella et al. 2019; Berkel et al. 2021) and even resulted in an improved disease-free survival (Trépanier et al. 2019). Additionally, a recently published paper concluded that a prehabilitation programme has turned out to be cost-effective with regard to health-care costs (Barberan-Garcia et al. 2019).

Prehabilitation is often used in colorectal cancer (CRC) treatment since colorectal resection is associated with high morbidity rates (Govaert et al. 2015). Furthermore, the treatment interval (diagnosis to treatment) generally comprises several weeks (Molenaar et al. 2021). CRC slowly develops over time, and treatment initiation is less hasty than in other types of cancer (Armaghany et al. 2012). In the Netherlands, the government recommends to initiate treatment within 5 weeks after pathological confirmation of CRC diagnosis in 80% of the patients and within 7 weeks in 100% (De Treeknormen Curatieve Zorg. 2020). This interval could optimally be used to proceed through a multimodal prehabilitation programme.

Awaiting more conclusive evidence about its effectiveness from ongoing trials, prehabilitation is already being implemented. There is consensus that prehabilitation should ideally be multimodal, containing various interventions such as exercise, nutritional support and protein supplementation, smoking and alcohol cessation, anaemia correction and psychological support (Scheede-Bergdahl et al. 2019). Beside the multimodal design, there is no (inter)national consensus on the content and design of such a programme. Furthermore, the implementation of a multidisciplinary programme proved to be difficult in practice as it involves many professionals and may be accompanied by logistic and financial difficulties.

Increased awareness of the potential benefit of prehabilitation has led to the recognition of the need to facilitate prehabilitation in Dutch hospitals. The ultimate goal of leading stakeholders (e.g. the Dutch Society for Surgery, the Dutch Healthcare Authority and insurance companies) is to implement a national and uniform evidence-based protocol. To evaluate the current daily practice in the Netherlands, we conducted a national survey among CRC surgeons with the aims of (1) assessing preoperative screening in general to enable distinction from interventions as part of a prehabilitation programme, (2) evaluating surgeons’ knowledge and opinions on prehabilitation and (3) collecting information on the general design of prehabilitation programmes in the Netherlands.

## Methods

### Study design and data collection

This study was conducted by the Department of Surgery of Máxima MC (MMC), a large teaching hospital in Veldhoven, the Netherlands. MMC is experienced in the conduct of research on prehabilitation (PREHAB trial: international, multicentre randomised controlled trial for patients with CRC (Netherlands Trial Register NL5784); non-randomised pilot study for patients with non-small cell lung cancer (Netherlands Trial Register NL8080)). Furthermore, MMC has implemented multimodal prehabilitation programmes in several cancer care pathways.

An online electronic survey (see Additional file 1) was developed by the authors and included questions about (1) the general use of preoperative screening, (2) the surgeon’s knowledge and opinions on prehabilitation and (3) the general design of prehabilitation programmes in the surgeon’s hospital. Additionally, colorectal surgeons were asked if they were interested in implementing prehabilitation in their hospital and if they would accept a potential delay of surgery for that purpose. The survey was built using the online survey software of SurveyMonkey^©^ (SVMK Inc., San Mateo, CA, USA). An answer to each question was mandatory before the participant could proceed to the next. All questions required multiple-choice or checkbox answers. Preoperative screening and prehabilitation domains were predefined by the authors and included nutritional status, frailty (according to the Geriatric 8 (G8) screening tool (Bellera et al. 2012)), physical status, mental health status, intoxications, anaemia and polypharmacy. In case other domains were used, participants were asked to elaborate on their answers. The survey was tested by three independent colorectal surgeons of MMC and was adjusted based on the feedback received. The dataset generated and analysed during the current study is available from the corresponding author on request.

### Study setting and population

In 2020, approximately 17.4 million inhabitants lived in the Netherlands with a life expectancy of 81.4 years, which is slightly higher compared to the mean life expectancy in Europe (Population - the Netherlands 2022; Life expectancy - the Netherlands 2022). In total, 143 general, academic, private and paediatric hospitals were located throughout the Netherlands, with 2.7 million admissions in 2020 (Hospital admissions - the Netherlands 2022). Hospital-related expenses comprised approximately 29.1 billion euros in 2019 (Hospital expenditure - the Netherlands 2022). Medical insurance is mandatory and inhabitants are generally registered with a local general practitioner, who serves as a gatekeeper.

The incidence of CRC is relatively high in the Netherlands compared to other European countries, with 60.8 new cases per 10,000 inhabitants (Colorectal cancer - the Netherlands 2022). In 2019, 6511 patients with colonic cancer and 2621 patients with cancer located in the rectum underwent surgical resection, mainly laparoscopically or robotic assisted (colon cancer: 82.3%; rectal cancer: 79%) (ColoRectal audit (DCRA) annual report 2019).

In the Netherlands, preoperative assessment prior to surgery is standard care. Once the indication for surgery is made, patients are referred to the outpatient preoperative screening clinic. Routine preoperative screening generally comprises of risk assessment (e.g. American Society of Anaesthesiologists classification (ASA); metabolic equivalent of task score (METs)); nutritional assessment (e.g. short nutritional assessment questionnaire (SNAQ), malnutrition universal screening tool (MUST)); routine blood work (e.g. haemoglobin, kidney function); and frailty screening (e.g. G8, Fried frailty index (FFI)). Furthermore, enhanced recovery after surgery (ERAS) programmes have been implemented in the majority of hospitals.

All regular Dutch hospitals offering CRC surgery were included. The study population comprised of one oncological surgeon per hospital. We used our own network to select participants. Based on our information, these surgeons were considered to be experts in the field of CRC surgery and were involved and well aware of their hospital’s policy. Nevertheless, in case a surgeon indicated that he or she was not suited to participate in our study, we intended to approach a second surgeon. We selected all Dutch academic, teaching and nonteaching hospitals for the current study. All surgeons received the first invitation for the survey in July 2020 and received subsequent reminders between August and October 2020. Results were handled anonymously. Ethical approval was deemed not necessary for this study.

### Statistical analyses

Data were analysed using SPSS version 22 (SPSS Inc. Chicago, IL, USA). Descriptive statistics were used to present data. Qualitative analyses were performed for data from comments or open questions.

## Results

### Participation rate

**Fig. 1:**
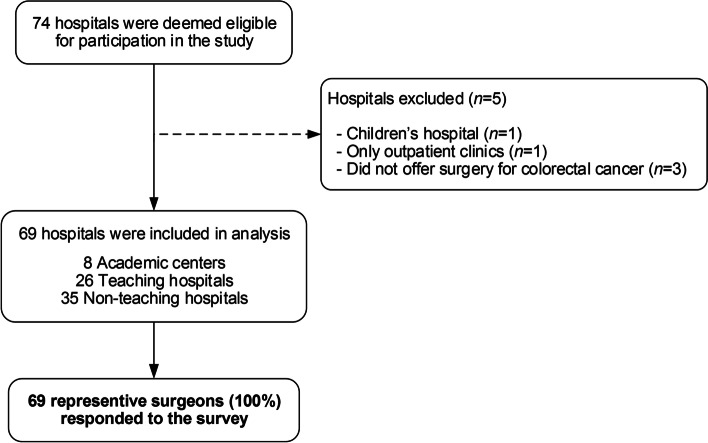
Flow diagram of included Dutch hospitals

Seventy-four hospitals were deemed eligible for participation in this study. Two hospitals were excluded because they were either children’s hospitals or outpatient clinics. Three hospitals were excluded because surgery for CRC was not being offered. This resulted in 69 unique hospitals (Fig. 1). A total of 69 representative surgeons responded to the survey resulting in a response rate of 100%. Forty-three surgeons responded after the first invitation with the remaining 26 surgeons responding after a reminder.

### Preoperative screening

**Fig. 2:**
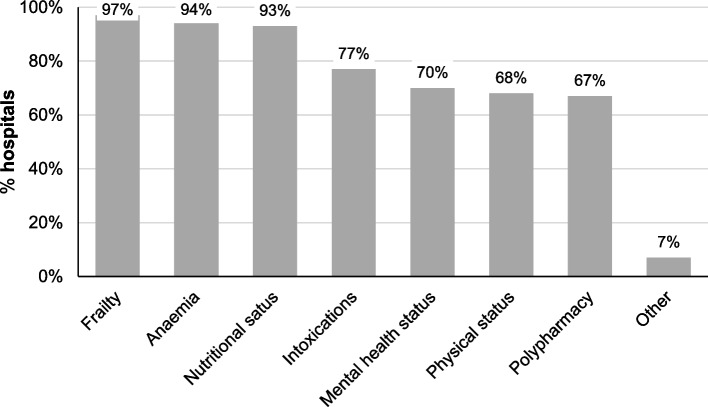
Current practices on preoperative screening for patients with colorectal cancer in the Netherlands, according to respondents (*n* = 69)

The use of preoperative screening in patients with CRC in various domains is presented in Fig. 2. Screening for nutritional status, frailty and anaemia was implemented in nearly all Dutch hospitals. Additional domains reported by the respondents included fall risk and geriatric assessment, evaluation of the patient’s domestic environment, comorbidity assessment and evaluation in general by the surgeon in the outpatient clinic. When preoperative screening revealed deficiencies in the screened domains, interventions were applied by default to address these deficiencies in only half of the hospitals (*n* = 34, 48.6%).

### The surgeon’s knowledge and opinion on prehabilitation

The term prehabilitation was understood by 98.6% of the respondents, and the majority (82.6%) believed a prehabilitation programme should have a multimodal design (Table 1). According to 53.6% of the respondents, multimodal was defined as at least two interventions. Several respondents suggested another definition of multimodal being as many interventions as needed based on the patient’s baseline assessment.

**Table 1:** General questions regarding prehabilitation, according to respondents (*n* = 69)

	***N* (%)**
**Are you familiar with the term “prehabilitation”?**	
Yes	68 (98.6%)
No	1 (1.4%)
**What do you mean by prehabilitation?**	
Any intervention	10 (14.5%)
Multimodal programme	57 (82.6%)
Other	1 (1.4%)
Missing^a^	1 (1.4%)
**What do you mean by multimodal?**	
At least two interventions	37 (53.6%)
At least three interventions	14 (20.3%)
Other	17 (24.6%)
Missing^a^	1 (1.4%)

^a^One respondent did not complete the survey and accounts for the missing value

### Current practice: the global design of prehabilitation programmes

Displayed in Table 2, a total of 46 Dutch hospitals (66.7%) had implemented some form of prehabilitation for patients scheduled for CRC surgery. Twenty-two hospitals (31.9%) had not implemented a prehabilitation programme for CRC at the time the survey was conducted. Of these, twenty hospitals (91%) were intending or willing to adopt such a programme, and only two respondents declared that they do not intend to implement a prehabilitation programme since they were not convinced by the benefits of this concept.

**Table 2:** The global design of prehabilitation programmes for patients with colorectal cancer

	Hospitals that provide prehabilitation (*n* = 46)	Hospitals that are interested in the implementation of prehabilitation (*n* = 20)
	***N* (%)**	***N* (%)**
**Do you/would you triage patients before start of a prehabilitation programme?**		
Yes	37 (80.4%)	18 (90.0%)
No	9 (19.6%)	2 (10.0%)
**What (sub-)groups (would) qualify for prehabilitation?**^**a**^		
All patients with CRC	8 (17.4%)	7 (35.0%)
Frail patients with CRC	33 (71.7%)	13 (65.0%)
Elder patients with CRC	19 (41.3%)	9 (45.0%)
High-risk patients with CRC	26 (56.5%)	11 (55.0%)
Patients with diseases other than CRC	9 (19.6%)	8 (40.0%)
**What domains are/would be included in the hospital’s prehabilitation programme for CRC?**^b^		
Nutritional status	45 (97.8%)	20 (100%)
Frailty	39 (84.8%)	16 (80.0%)
Physical status	39 (84.8%)	18 (90.0%)
Mental status	22 (47.8%)	15 (75.0%)
Intoxications	27 (58.7%)	15 (75.0%)
Anaemia	38 (82.6%)	20 (100%)
Polypharmacy	19 (41.3%)	14 (70.0%)
Other	1 (2.2%)	1 (5.0%)
**What is/would be the design of the interventions?**^**a**^		
Advices for home	28 (60.9%)	9 (45.0%)
A structured, standardised programme equal for all patients	6 (13.0%)	0 (0.0%)
A structured, individualised tailored programme	34 (73.9%)	20 (100%)
Other	11 (23.9%)	0 (0.0%)
**In what setting is/would the programme being/be offered?**^**a**^		
Hospital based	24 (52.2%)	6 (30.0%)
In primary care facilities	39 (84.8%)	19 (95.0%)
In the gym	14 (30.4%)	6 (30.0%)
Home based	32 (69.6%)	16 (80.0%)

Abbreviations: *CRC*, colorectal cancer; *N*, number of respondents

^a^Three hospitals not included. Two respondents were not intending to implement prehabilitation within the near future and one is missing

^b^Check box questions; more than one answer allowed

The majority of hospitals offered prehabilitation to more than one subgroup with elderly, frail patients being targeted most often. Only 17.4% of the hospitals did not focus on subgroups and offered prehabilitation to all patients with CRC. All prehabilitation programmes included more than one intervention. Polypharmacy and mental health status were underrepresented domains in the programmes. The interventions were offered both for at home as well as a structured, individualised programme tailored for the individual patient in 26 (56.5%) hospitals. Eleven surgeons (23.9%) responded that the programme was not structured or standardised but consisted of individualised interventions based on the patient’s needs. The majority of the interventions were being outsourced to primary care facilities.

### Treatment interval

**Fig. 3:**
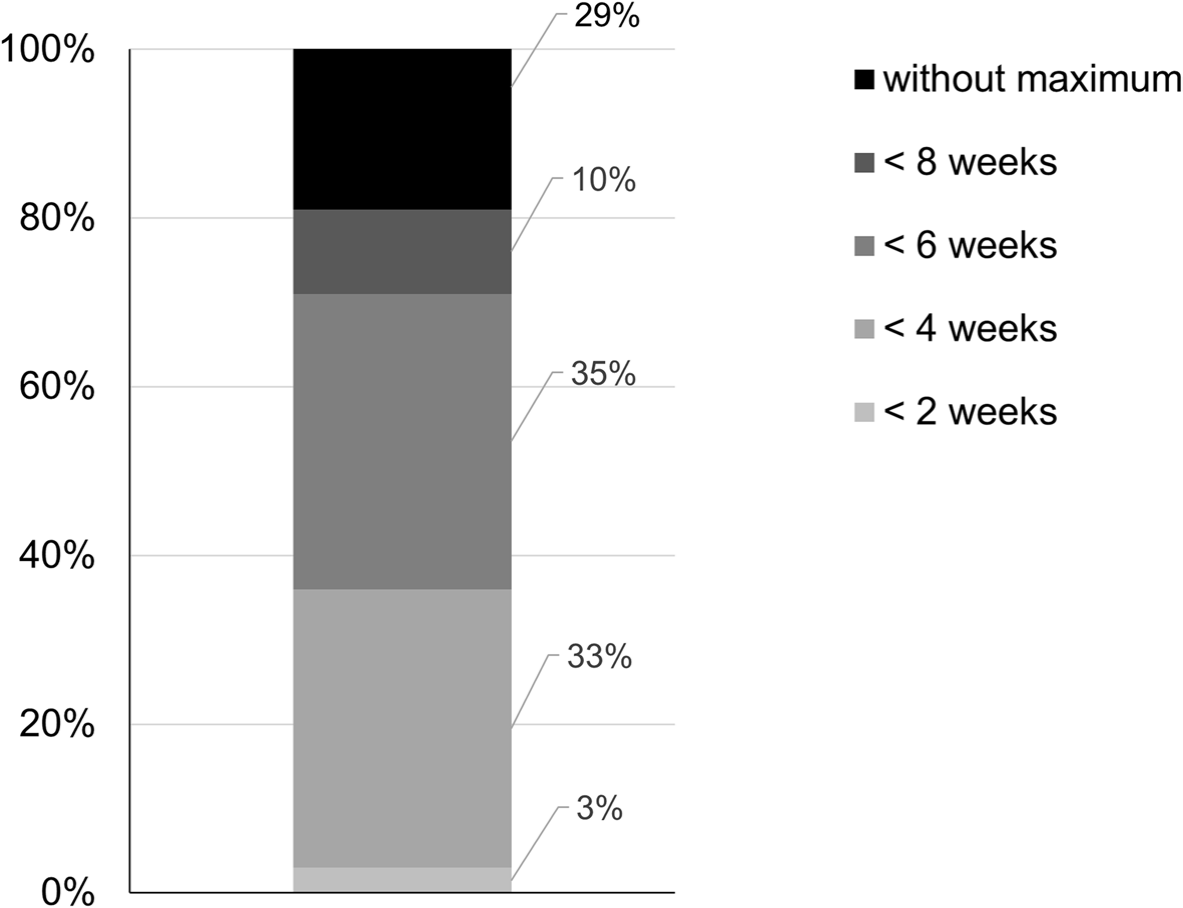
Opinion about interval from diagnosis until surgical treatment to facilitate prehabilitation

Finally, we had asked the surgeons whether or not they were willing to extend time from pathological diagnosis to surgery (treatment interval) in order to facilitate prehabilitation. Nearly half of the respondents were willing to perform surgery after a maximum of 6 weeks or 8 weeks after diagnosis, and 29.0% of the respondents was willing to extend time to surgery as long as necessary to optimise the patient’s condition (Fig. 3).

## Discussion

This study assessed the current state of prehabilitation for patients with CRC in Dutch hospitals. Preoperative screening is sufficiently incorporated in Dutch hospitals. However, it is remarkable that this does not translate into acting on deficiencies accordingly and, moreover, to interventions in the preoperative period that improve the patient’s condition. In the Netherlands, the concept of prehabilitation has nowadays been incorporated by 67% of the hospitals, but there is high variability in the design of the programme and the setting in which prehabilitation is being offered.

Prehabilitation is an upcoming phenomenon, a concept rapidly embraced by health-care facilities worldwide. In general, preoperative optimisation consists of screening, assessment and interventions (Minnella and Carli 2018; Bates et al. 2020) addressing modifiable risk factors with the aim to improve the patient’s condition. Main domains in the programme focused on physical, nutritional and mental health status (Minnella and Carli 2018). Additionally, addressing lifestyle behaviour and, in CRC, anaemia correction may be included in the programme. Combining interventions in a multimodal programme results in a synergistic effect (Scheede-Bergdahl et al. 2019).

### Preoperative screening

Factors that are traditionally being screened in the preoperative period such as age, gender and ASA classification cannot be optimised. In contrast, risk factors that are modifiable have been studied thoroughly (Rooijen et al. 2017; McDermott et al. 2015). The present study shows that current preoperative screening methods in the Netherlands include various domains. The high screening rates for frailty and malnutrition can be explained by the fact that these domains are imposed by a national safety programme focused on the frail elderly (The Dutch Safety Management System and (Veiligheids Managements Systeem). (2009)). Additionally, haemoglobin is routinely being screened according to the 4–5-6 rule (haemoglobin levels of 4, 5 and 6 mmol/L) included in the Dutch blood transfusion guideline (National Users’ Board Sanquin Blood Supply 2011). However, this rule does not take into account the underlying causes of anaemia and correction thereof. Despite current recommendations of anaemia management in the latest ERAS guidelines (Gustafsson et al. 2019), translation into clinical practice is still inconvenient (Wilson et al. 2018). A recently published study by Bosker et al. reported preoperative anaemia in approximately 20% of the Dutch patients undergoing right-sided colonic resection and a similar amount receiving blood transfusion postoperatively (Bosker et al. 2019). Since anaemia is a major risk factor for postoperative complications, e.g. colorectal anastomotic leakage (Huisman et al. 2020) and worse survival (Kwon et al. 2020; Bruns et al. 2019), preoperative optimisation is of great importance. Without an intervention, screening of risk factors like this is futile.

### Prehabilitation in Dutch hospitals

The results from this study revealed that nearly all respondents (97%) were familiar with the concept. Some form of prehabilitation has already been implemented in two-thirds of the Dutch hospitals. As mentioned before, the programme should be multimodal. This was also clearly reported by our respondents (82.6%). The majority of the programmes were carried out in primary care facilities. Even though supervision is provided in both in-hospital and primary care setting, the latter minimises the potential travel burden. However, for high-risk patients, for example, in-hospital exercise may be more appropriate. In the near future, triage will enable professionals to distinguish subgroups with an indication for in-hospital, primary care or home-based exercises.

### Logistical and financial challenges

There are several challenges regarding the implementation of prehabilitation in routine clinical practice, and financial factors should be taken into account. Prehabilitation is not included in the health insurance policy in the Netherlands. Furthermore, implementing a prehabilitation programme is time-consuming. The multimodal design of the programme involves many disciplines, and all departments have their own board of directors and own budget. Redesigning the care pathway with a programme that suits all departments is challenging. More importantly, in order to minimise the burden for patients, departments need to cooperate quickly and accurately in order to harmonise all appointments for the patient. We would therefore advise hospitals with the intention to implement such a programme to adjust and adopt one of the protocols that already have been published (Rooijen et al. 2019).

### Interventions to improve mental health status

A domain underrepresented in current prehabilitation programmes in the Netherlands is mental health status. From a practical perspective, this domain is as important as the nutritional and/or exercise intervention since a good mental health status (and consequently motivation) is necessary to complete an intensive programme. However, it remains unclear from the existing evidence which psychological interventions are most effective with regard to surgical outcomes (postoperative complications, length of stay, mortality) or patient-reported outcomes (Levett and Grimmett 2019). Optimal information provision, maximal involvement of the patient within their own cancer pathway, relaxation techniques and cognitive therapy (insight in the patient’s coping strategy) may form the base for psychological interventions. While it may seem intuitive to mentally prepare patients before surgery, interventions are difficult to design. When indicated, intensive guidance by a trained psychologist may be beneficial.

### Treatment interval

The majority of the respondents (34.8%) was willing to postpone surgery with a maximum of 6 weeks after pathological confirmation of diagnosis to optimise the patient’s condition with prehabilitation. Furthermore, 29.0% of the respondents stated that for an individual patient, when necessary, there should be no limit to the period needed to optimise the patient’s condition. A recently published systematic review concluded that there is a safe window to prehabilitate patients with colon cancer within 8 weeks from diagnosis (Molenaar et al. 2021).

### Limitations

One of the key strengths of this study is that we have collected a comprehensive range of data (e.g. opinions, beliefs and values) from all hospitals in the Netherlands that perform CRC surgery. In order to get a complete overview, open answers were allowed when no other option would suffice. It was difficult to organise the answers and to capture the actual practice regarding content and design of prehabilitation programmes for some hospitals. We chose to collect responses of only one surgeon performing surgery for CRC to represent his/her hospital in order to prevent a high variety in responses within hospitals. We expected the responses of surgeons who were aware of the details regarding the hospitals’ policy on prehabilitation to be more accurate than the information of surgeons that were selected randomly. Moreover, assessment of the consensus of different healthcare providers within hospitals was not one of our goals. However, this might limit the validity of the responses and might introduce bias. Another limitation is that in order to limit the length of the survey, details of the programmes on measures and tests for screening and assessment were not included. Furthermore, not all modifiable risk factors, such as glycaemic status, were included in the survey. However, including those variables in the current study would perhaps have resulted in cluttered results but are beyond the scope of this study. Even though this survey was designed to evaluate the current clinical practice regarding prehabilitation in the Netherlands, our findings could be of interest for other countries worldwide.

## Conclusions

In conclusion, this study presents a high variability in prehabilitation practices in CRC care. Whereas preoperative screening is sufficiently incorporated in Dutch hospitals, optimising patients in the context of multimodal prehabilitation seems to be challenging. Uniform clinical guidelines of such programmes enable comparison and further implementation of prehabilitation programmes across all countries.

## Supplementary Information

**Additional file 1: Appendix A.** The national survey about prehabilitation for patients with colorectal cancer.

## Data Availability

Please contact author for data requests.

## Abbreviations

ASA: American Society of Anaesthesiologists
CRC: Colorectal cancer
ERAS: Enhanced recovery after surgery
FFI: Fried frailty index
G8: Geriatric 8 frailty screening tool
METs: Metabolic equivalent of task score
MMC: Máxima Medical Centre
MUST: Malnutrition Universal Screening Tool
SNAQ: Short Nutritional Assessment Questionnaire
