# Supplementary material for: Prehabilitation for patients with colorectal cancer: a snapshot of current daily practice in Dutch hospitals
Source: Perioper Med (Lond). 2023 May 8;12:15. doi: 10.1186/s13741-023-00299-y (PMC10165784; doi:10.1186/s13741-023-00299-y)
Supplement: Supplementary file 1 — Additional file 1: Appendix A. The national survey about prehabilitation for patients with colorectal cancer. [file 13741_2023_299_MOESM1_ESM.docx]

**Appendix A**

**The national survey about prehabilitation for patients with colorectal cancer sent to Dutch colorectal surgeons in all hospitals**

1. What is the name of your hospital? *The purpose of this question is to document responses. This information will be handled strictly confidential and won’t be used for publication.*

**Preoperative optimisation**

*“The next part is about the preoperative work-up of patients with CRC in your hospital. Specifically, it is about preoperative screening and intervention(s) as part of standard care.”*

1. Does your hospital screen patients with CRC preoperatively as part of standard care on the following domains? (*multiple selections possible)*

- Nutritional status
- Frailty
- Physical status
- Mental status
- Intoxications
- Anaemia
- Polypharmacy
- Other:

1. Is an intervention applied when scores on a particular domain are insufficient? *Example of interventions include but are not limited to the following domains: nutritional status, frailty, physical status, mental status, intoxications, anaemia, polypharmacy.*

- Yes, an intervention is always applied whenever this is deemed necessary regarding one or more domains
- An intervention is not always applied even though results are insufficient based on screening of one or more domains
- An intervention is not applied

**Prehabilitation**

1. Are you familiar with the term “prehabilitation”?

- Yes
- No

If yes, “*there is no universally accepted definition of prehabilitation. Large variations of content and design of prehabilitation programmes have been reported in the literature. Clearly, there is need for a generally accepted definition and we are interested in your opinion.”*

If no, “*the term prehabilitation refers to interventions in the preoperative period to improve functional capacity prior to surgery and consequently, improve outcome and fasten recovery postoperatively. Prehabilitation often consists of a multimodal, structured program. Examples include but are not limited to the following: nutritional support, smoking cessation, mental support, anaemia correction.”*

1. What do you mean by prehabilitation?

- Any preoperative intervention for optimisation
- A structured, preoperative multimodal programme for optimisation
- Other:

1. What do you mean by multimodal? *Examples include but are not limited to the following: nutritional support, smoking cessation, mental support, anaemia correction.*

- At least two interventions
- At least three interventions
- Other:

1. Does your hospital offer a prehabilitation programme to patients with CRC?

- Yes, *please go to question 8*
- No, *please go to question 13*

***“The next part is about prehabilitation of patients with CRC in your hospital.”***

1. What (sub)groups qualify for prehabilitation? (*multiple selections possible)*

- All patients with CRC
- Frail patients with CRC
- Elder patients with CRC
- High-risk patients with CRC
- Patients with diseases other than CRC

1. Do you triage patients before start of a prehabilitation programme?

- Yes
- No

1. What domains are included in the hospital’s prehabilitation programme for CRC? (*multiple selections possible)*

- Nutritional status
- Frailty
- Physical status
- Mental status
- Intoxications
- Anaemia
- Polypharmacy
- Other:

1. What is the design of the interventions? (*multiple selections possible)*

- Advice for home
- A structured, standardised programme equal to all patients
- A structured, individualised programme tailored for each patient
- Other:

1. In what setting is the programme being offered? (*multiple selections possible)*

- Hospital-based, *please go to question 21*
- In primary care facilities, *please go to question 21*
- In the gym, *please go to question 21*
- Home-based, *please go to question 21*

1. What is the reason that you are not providing prehabilitation for patients with CRC in your hospital?

- We never thought of that
- Due to logistic reasons
- Due to financial reasons
- Other:

1. Would you like to offer a prehabilitation programme to patients with CRC?

- Yes, *please go to question 16*
- No

1. What is the reason not wanting to offer prehabilitation to patients with CRC? (*multiple selections possible)*

- Due to financial reasons
- Due to logistic reasons
- I could not convince my colleagues of the benefit
- I am not convinced of the benefit
- Other:

1. What (sub)groups would qualify for prehabilitation? (*multiple selections possible)*

- All patients with CRC
- Frail patients with CRC
- Elder patients with CRC
- High-risk patients with CRC
- Patients with diseases other than CRC

1. Would you triage patients with CRC before the start of a prehabilitation programme?

- Yes
- No

1. What domains would be included in the hospital’s prehabilitation programme for CRC? (*multiple selections possible)*

- Nutritional status
- Frailty
- Physical status
- Mental status
- Intoxications
- Anaemia
- Polypharmacy
- Other:

1. What would be the design of the interventions? (*multiple selections possible)*

- Advice for home
- A structured, standardised programme equal to all patients
- A structured, individualised programme tailored for each patient
- Other:

1. In what setting would the programme be offered? (*multiple selections possible)*

- Hospital-based
- In primary care facilities
- In the gym
- Home-based

1. How should prehabilitation be financed?

- Patients finance their own prehabilitation
- The insurance companies reimburse the costs
- The hospitals finance the programme
- Other:

*“According to national guidelines, the time interval between diagnosis of CRC and surgical treatment is five to six weeks in the Netherlands. This time can be used for prehabilitation.”*

1. Would you postpone surgery in order to optimise the patient’s condition preoperatively?

- Yes, up to a maximum of two weeks from pathologic diagnosis
- Yes, up to a maximum of four weeks from pathologic diagnosis
- Yes, up to a maximum of six weeks from pathologic diagnosis
- Yes, up to a maximum of eight weeks from pathologic diagnosis
- Yes, as long as it takes
- No, I would not postpone the surgery
